# The effect of pre- and post-operative physical activity on recovery after colorectal cancer surgery (PHYSSURG-C): study protocol for a randomised controlled trial

**DOI:** 10.1186/s13063-017-1949-9

**Published:** 2017-05-08

**Authors:** Aron Onerup, Eva Angenete, David Bock, Mats Börjesson, Monika Fagevik Olsén, Elin Grybäck Gillheimer, Stefan Skullman, Sven-Egron Thörn, Eva Haglind, Hanna Nilsson

**Affiliations:** 1 Scandinavian Surgical Outcomes Research Group (SSORG), Department of Surgery, Institute of Clinical Sciences, Sahlgrenska Academy, University of Gothenburg, Gothenburg, Sweden; 2 Department of Neuroscience and Physiology, Sahlgrenska Academy, University of Gothenburg, Gothenburg, Sweden; 3 Department of Food and Nutrition and Sport Science, University of Gothenburg, Gothenburg, Sweden; 4 Sahlgrenska University Hospital/Östra, Gothenburg, Sweden; 5 Department of Gastrosurgical Research and Education, Sahlgrenska Academy at Gothenburg University, Gothenburg, Sweden; 6 Department of Physical Therapy and Surgery, Sahlgrenska University Hospital, Gothenburg, Sweden; 7 Department of Surgery, Skövde Hospital/KSS, Skövde, Sweden; 8 Department of Anesthesiology, Institute of Clinical Sciences, Sahlgrenska Academy, University of Gothenburg, Gothenburg, Sweden

**Keywords:** Physical activity, Inspiratory muscle training, Post-operative recovery, Colorectal cancer surgery

## Abstract

**Background:**

Surgery for colorectal cancer is associated with a high risk of post-operative adverse events, re-operations and a prolonged post-operative recovery. Previously, the effect of prehabilitation (pre-operative physical activity) has been studied for different types of surgery, including colorectal surgery. However, the trials on colorectal surgery have been of limited methodological quality and size. The aim of this trial is to compare the effect of a combined pre- and post-operative intervention of moderate aerobic physical activity and inspiratory muscle training (IMT) with standard care on post-operative recovery after surgery for colorectal cancer.

**Methods/design:**

We are conducting a randomised, controlled, parallel-group, open-label, multi-centre trial with physical recovery within 4 weeks after cancer surgery as the primary endpoint. Some 640 patients planned for surgery for colorectal cancer will be enrolled. The intervention consists of pre- and post-operative physical activity with increased daily aerobic activity of moderate intensity as well as IMT. In the control group, patients will be advised to continue their normal daily exercise routine. The primary outcome is patient-reported physical recovery 4 weeks post-operatively. Secondary outcomes are length of sick leave, complication rate and severity, length of hospital stay, re-admittances, re-operations, post-operative mental recovery, quality of life and mortality, as well as changes in insulin-like growth factor 1 and insulin-like growth factor-binding protein 3, perception of pain and a health economic analysis.

**Discussion:**

An increase in moderate-intensity aerobic physical activity is a safe, cheap and feasible intervention that would be possible to implement in standard care for patients with colorectal cancer. If shown to be effective, this lifestyle intervention could be a clinical parallel to pre-operative smoke cessation that has already been implemented with good clinical results.

**Trial registration:**

ClinicalTrials.gov identifier: NCT02299596. Registered on 17 November 2014.

**Electronic supplementary material:**

The online version of this article (doi:10.1186/s13063-017-1949-9) contains supplementary material, which is available to authorized users.

## Background

All surgical procedures are followed by a phase of post-operative recovery, which typically includes both individual distress and consumption of health care resources. Recovery after colon and rectal cancer surgery is known to include a high level of post-operative adverse events, with reported rates up to 35%, re-operation rates of around 10% and 30-day mortality of around 2% [1–3]. The Swedish National Board of Health and Welfare estimates that, without complications, most patients with colon cancer return to normal function within 4–6 weeks post-surgery, whereas rectal cancer patients require a longer time to recover, up to 6–8 weeks [4, 5].

Prospective observational studies have shown that physical activity before and after a colorectal cancer diagnosis as well as after treatment is associated with better survival [6–8]. This is plausible because the multiple effects of physical activity include effects on endurance, strength and functional capacity; mental and stress-related health and quality of life (QoL) [9]; immune function [10]; and cardiovascular morbidity and mortality [9]. Possibly contributing mediators of this effect include insulin-like growth factor (IGF)-1 and insulin-like growth factor-binding protein (IGFBP)-3 [11, 12]. Researchers in one small randomised controlled trial (RCT) reported effects of post-operative physical activity on the IGF axis with a significant increase in IGF-1 and IGFBP-3 in stages II–III colorectal cancer survivors [13], which could be of benefit for the patient.

In previous studies evaluating prehabilitation with pre-operative physical activity before colorectal cancer surgery [14–17], researchers reported effects on functional exercise capacity, but their studies did not have enough power to detect any effects on adverse events or recovery. In one study, researchers found positive effects on walking capacity in the group performing the ‘sham’ intervention, consisting of walking, compared with the group that performed more intense physical activity [14], probably partly due to lower compliance with more intense activity. Authors of a recent systematic review of prehabilitation before colorectal cancer surgery concluded that there is currently no clear evidence that an improvement in fitness translates into improved peri- and post-operative outcomes. It was further concluded that further adequately powered RCTs are needed to investigate whether pre-operative exercise improves post-operative morbidity and mortality [18]. Authors of another recent systematic review on prehabilitation and abdominal cancer surgery concluded that previous trials in this area suffer from methodological heterogeneity [19]. All trials in their systematic review had functional capacity measures as their primary outcome measures. In a third recent systematic review on prehabilitation and abdominal surgery, it was concluded that prehabilitation appears to decrease post-operative complications after abdominal surgery but that more high-quality studies are needed [20]. To summarise, there have been several trials assessing the effect of prehabilitation and abdominal surgery, but they have been of relatively small size, and their primary outcome measures have been to assess the effect on functional capacity measures rather than on outcome measures of clinical post-operative recovery.

There are other areas of surgery where there is evidence of positive effects from pre-operative physical activity (e.g., thoracic surgery) [21–24]. Inspiratory muscle training (IMT) is one type of physical activity that has been found to be successful in reducing post-operative pulmonary complications and hospital length of stay [22].

Our hypothesis to be tested in the present study is that individuals receiving an intervention aimed at increasing their pre- and post-operative level of physical activity of moderate intensity will experience faster post-operative recovery in comparison with standard care. The primary aim of this study is to investigate whether a training programme with intensified physical activity and IMT prior to and after surgery for colorectal cancer enhances post-operative recovery assessed as patient-reported physical recovery in comparison with control subjects. Secondary aims are to investigate the effect of the training programme on sick leave, adverse events, hospital length of stay, resumption of QoL, re-operations, re-admittances, post-operative mental recovery, mortality, IGF-1, IGFBP-3, experienced pain and health economics in comparison with control subjects.

## Methods/design

This study is a randomised, controlled, parallel-group, open-label, multi-centre trial. The primary endpoint is physical recovery 4 weeks after surgery. The locations of the study are university and county hospitals in Sweden that regularly perform colorectal surgery. At the moment, two hospitals participate in the study, but more centres may be added. A list of study sites can be obtained from the corresponding author. Eligible participants are all patients aged 20 years or older scheduled for elective colorectal cancer surgery in the participating hospitals during the inclusion period. Informed consent forms will be signed and dated prior to conduct of any study-specific procedures. Exclusion criteria are emergency surgery, local surgery such as transanal endoscopic microsurgery, cytoreductive surgery and subsequent hyperthermic intraperitoneal chemotherapy, as well as patients unable to understand information given or who are considered unable to perform study-specific procedures.

### Groups

The intervention and control groups will differ with regard to both pre- and post-operative care in the outpatient setting. During the hospital stay, both groups will be treated according to standard care. In the intervention group, patients perform the following intervention pre-operatively during 2 weeks ± 4 days:

- *Thirty minutes of daily aerobic exercise added to the normal daily exercise routine of each patient:* The type of aerobic exercise is chosen by the patient, and the exercise is performed in the home environment. The level of exercise should produce shortness of breath, but the patient should be able to talk without much effort, corresponding to relative medium-intensity activity for the individual, according to Borg’s Rating of Perceived Exertion Scale [25].
- *IMT:* The patient’s maximal inspiratory pressure (MIP) is assessed by a physiotherapist at residual volume with a MicroRPM respiratory pressure meter (CareFusion, Höchberg, Germany). The assessment is performed with the patient sitting, and the best result of at least three is noted [26]. The IMT is performed with a Threshold IMT device (Philips Respironics, Eindhoven, The Netherlands). Starting with a resistance of 30% of MIP, the patient is instructed on how the resistance can be increased if needed. The patient is instructed to perform 30 breaths two times, twice daily.

During hospitalisation post-surgery, patients in both groups receive standardised information from a physiotherapist starting on the first day after surgery, including breathing exercises with positive expiratory pressure by mouthpiece (open procedures) or deep breaths (laparoscopic procedures) every 2 h during the daytime, as well as early and frequent mobilisation. The including hospitals may adhere to different extents to the Enhanced Recovery After Surgery (ERAS) protocol [27], but they are instructed to treat all patients according to local routine, regardless of randomisation allocation. The hospital’s adherence to the ERAS protocol will be reported. Hospital staff are not actively informed about the patient’s allocation.

Post-operatively, from the time of discharge from hospital and for four weeks, patients in the intervention groups perform the following:

- *Thirty minutes of daily exercise added to the normal daily exercise routine of each patient:* The level of exercise should produce shortness of breath, but the patient should be able to talk without much effort [25].

A research nurse will contact patients in the intervention group by phone 1 week into the pre-operative intervention and 3 weeks into the post-operative intervention to ascertain that the physical activity and IMT are progressing as planned, with possibility for modifications. To improve and monitor adherence to the intervention, all patients in the intervention group fill in an exercise diary on a daily basis pre-operatively as well as up to 4 weeks after discharge from hospital, where instructions are clearly outlined for the intervention group. In the control group, patients will be advised to continue their normal daily exercise routine. Patients in the control group will receive a similar diary where they will register their physical activity, but without specified goals or instructions.

### Outcomes

The primary endpoint is physical recovery as measured by patient-reported experienced physical recovery. Patient-reported physical recovery will be assessed by questionnaires 4 weeks and 1 year post-operatively, with the question, “To what extent do you feel fully physically recovered”? Answering categories are 0%, 25%, 50%, 75% and 100%. This question has been validated face-to-face and has previously been used in several cohort studies [28–30].

Secondary endpoints are as follows:

- *Sick leave:* This will be measured by length and degree of sick leave, for all causes, during the first year post-operatively.
- *Adverse events classified according to the Clavien-Dindo classification of surgical complications* [31] *within the first 90 days post-operatively:* These will be reported as the number of complications grade O-IIIa and IIIb-V. We will also report information gained from the comprehensive complications index for all patients, which is derived from the Clavien-Dindo classification [32].
- *Length of hospital stay*
- *Post-operative recovery of QoL measured with several instruments pre-operatively and 4 weeks and 1 year post-operatively:* Quality-adjusted life-years (QALYs) will be possible to calculate because EQ-5D is included in the questionnaire [33].
- *Re-operations and re-admittances within the first 12 months after primary surgery:* All forms of surgery and all re-admissions to hospital will be registered.
- *Patient-reported experienced mental recovery 4 weeks and 12 months post-operatively, used in previous observational cohort studies* [28–30]
- *Post-operative all-cause and cancer-specific mortality, measured at 3 and 5 years*
- *Changes in IGF-1, IGFBP-3 and glycated haemoglobin (HbA1c) levels from baseline to time of surgery (IGF-1 and IGFBP-3) and 4–6 weeks after surgery (all):* These will be determined for a subgroup of the patients and analysed prior to the rest of the outcomes.
- *Post-operative pain measured with Brief Pain Inventory–Short Form (BPI-SF):* This will be measured 4 weeks and 12 months post-operatively [34].
- *Health economic analysis of resource consumption 12 months post-operatively:* This will be performed separately, depending on other outcomes.

### Questionnaires used in this study

The pre- and post-operative questionnaires (4 weeks and 12 months post-operatively) in this study are composed mainly of the following well-established questionnaires:

- *The International Physical Activity Questionnaire (IPAQ)* [35]*:* a validated questionnaire for assessing level of physical activity that consists of seven questions
- *Saltin-Grimby Physical Activity Level Scale* [36]*:* A single question that has been shown to have high validity and reliability, being associated with cardiovascular risk factors [37, 38], morbidity [39, 40] and mortality [41]
- *RAND-36, also known as the 36-item Short Form Health Survey, or SF-36* [42]*:* A health profile survey with high clinical validity [43] that consists of 11 questions with several sub-questions and a total of 36 items
- *Health-related QoL:* Assessed with the well-established form EQ-5D [44], which consists of six questions; this can also serve as a basis for calculating QALYs [33]
- *Sense of coherence:* Questions for assessing how individuals experience themselves in relation to their environment [45], assessed with 29 questions
- *Alcohol Use Disorders Identification Test alcohol consumption questions (AUDIT-C)* [46]*:* Alcohol use is assessed using this instrument, which is a screening tool for problem drinking and consists of three questions.
- *Pain:* Assessed using the BPI-SF questionnaire [34], consisting of 15 questions regarding different aspects of pain

### Allocation

A research nurse will perform enrolment and the electronic randomisation. The trial is an open-label study because neither patients nor research nurses nor physiotherapists can be blinded. However, registration regarding complications, hospital length of stay, re-operations, re-admittances, mortality and sick leave will be blinded. The nurses and surgeons responsible for providing care during the hospital stay are not actively informed about allocation. Randomisation will be performed as block randomisation with a 1:1 allocation and a block size that will be unknown to study personnel. To ensure good balance of participant characteristics in each group, randomisation will be stratified with regard to surgical method (laparoscopic versus open), tumour site and pre-operative treatment (colon, rectum with/without radiotherapy), and study centre. Randomisation numbers (patient numbers) will be assigned strictly sequentially as patients become eligible for randomisation. The assigned group is automatically generated and cannot be changed retrospectively.

The screening log and randomisation process is part of an electronic clinical record form in which inclusion and exclusion criteria are noted, as well as information needed for stratification. All patients planned for elective colorectal surgery at participating hospitals are included in the screening log. Patients excluded will be included in the screening log with information on age, sex and reason for exclusion.

### Sample size

On the basis of previous observational data on recovery after surgery for colon cancer in Swedish patients, an initial sample size was calculated as 370 patients. Previous knowledge about the possible effect of this type of physical activity is scarce, however, and the assumptions underlying the sample size were associated with uncertainty. An interim sample size recalculation was decided to be performed after 6 months of follow-up data were available for the first 100 patients. The aim of the analysis was to obtain preliminary estimates of the rates of the two primary outcome measures—patient-reported physical recovery and sick leave—in the groups, as well as an effect size to enable a sample size re-estimation.

An external independent data monitoring committee consisting of two physicians and a statistician performed the interim analysis on data where information about the true type of intervention was concealed. After 100 patients were included, 77 patients had data on physical recovery 4 weeks post-operatively, with 49% and 37% feeling highly physically recovered in the two groups (i.e., a relative difference of 32%). For true rates of these magnitudes, a difference will be detected with 80% power using a total of 640 patients and a two-sided test with a 2.5% significance level (Bonferroni adjustment for interim look). After the interim analysis, sick leave was no longer considered a primary outcome measure, and physical recovery remained the sole primary outcome measure. The study is planned to continue until 640 patients are included, given the observed frequency of withdrawn consent and other reasons for discontinuation.

Given the nature of the intervention, with no anticipated serious side effects, and with patients generally willing to do what they can to improve their cancer prognosis, we assume that the rate of enrolment of patients will be high. All medical staff working in participating departments of surgery will receive information about the study to further enhance possibilities of adequate participant enrolment. All patients planned for colorectal cancer surgery at participating hospitals will receive a telephone call with oral information as well as written information about the study before their pre-operative visit, as well as oral information provided by a research nurse at their pre-operative outpatient visit.

### Data collection

Data collection will be performed at the following times:

- The pre-operative case report form (CRF) is filled out by a nurse using data collected at inclusion. This includes characteristics of importance for post-operative recovery, such as weight; length; and basic clinical information such as haemoglobin, leucocyte count, C-reactive protein (CRP), serum albumin, serum creatinine and analyses of circulating levels of IGF-1, IGFBP-3 and HbA1c (*see* below). It also includes data on pre-operative MIP (measured as centimetres of water) for patients in the intervention group.
- IGF-1 and IGFBP-3 will be analysed at inclusion, before the start of anaesthesia and 4–6 weeks post-operatively in a sub-group of patients. IGF-1 will be analysed using an IDS-iSYS (Immunodiagnostic Systems, Tyne & Wear, UK), and IGFBP-3 will be analysed using an IMMULITE 2000 XPi (Siemens Medical Solutions, Malvern, PA, USA). HbA1c will be analysed before the intervention and 4–6 weeks post-operatively in a sub-group of approximately 80 patients at one hospital. Analysis of HbA1c is conducted by high-performance liquid chromatography (ion exchange liquid chromatography) with the Mono-S 5/50 GL Tricorn column (GE Healthcare Bio-Sciences, Uppsala, Sweden).
- At discharge from hospital, a CRF is filled out with information on hospital length of stay, possible time of stay in the intensive care unit, blood transfusions, post-operative haemoglobin, leucocyte count, CRP and destination after discharge.
- Patient files will be examined to classify adverse events according to the Clavien-Dindo classification of surgical complications [47] for the first 90 days post-operatively by one or two doctors per site without access to group denomination for each patient.
- All included patients will be asked to fill out questionnaires at inclusion, 4 weeks post-operatively and 12 months post-operatively. These will include the following validated questionnaires: IPAQ [35], Saltin-Grimby Physical Activity Level Scale [36], RAND-36 [42], EQ-5D [44], sense of coherence [48], general QoL questions from previously used questionnaires [49, 50], AUDIT-C [46], BPI-SF [34], pack-years of smoking and questions about patients’ perceived level of recovery, re-admittances and re-operations.
- Data will be retrieved from the Swedish Social Insurance Agency regarding length, degree and reason for sick leave during the first 12 post-operative months. Patients in occupation or seeking work will be offered 3 weeks of sick leave at hospital discharge. A prolongation by 1 week at a time until visit at the outpatient clinic will be granted at the request of the patient by way of a telephone call to an answering machine.
- Clinical data and TNM classification will be retrieved from the Swedish National Colorectal Cancer Registry, in addition to data on mortality 3 and 5 years post-operatively.
- Complications and adverse events due to the intervention will be registered and analysed.

**Fig. 1:**
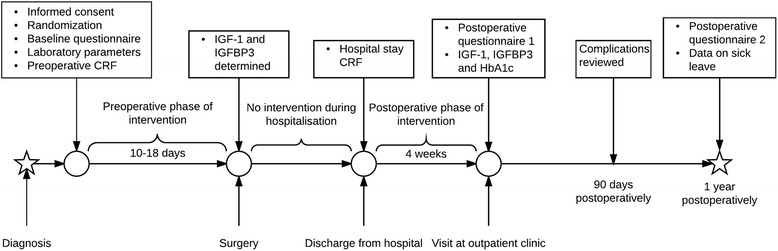
Participant time line. Time schedule of enrolment, intervention, assessments and visits for participants. *CRF* Case report form, *HbA1c* Glycated haemoglobin, *IGF-1* Insulin-like growth factor 1, *IGFBP-3* Insulin-like growth factor-binding protein 3

**Fig. 2:**
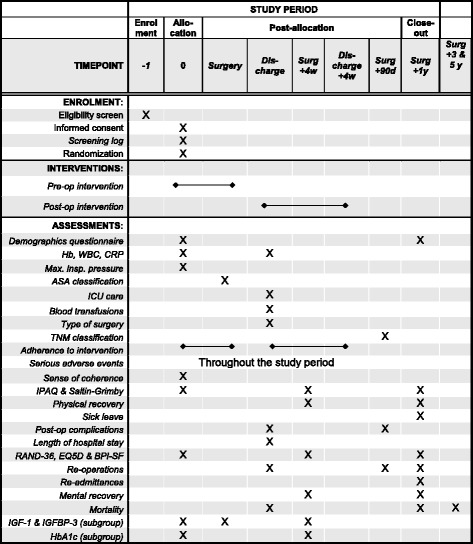
Standard Protocol Items: Recommendations for Interventional Trials (SPIRIT) figure showing important events in the trial and their respective time points. *Hb* Haemoglobin, *WBC* White blood cells, *ASA* American Society of Anesthesiologists physical status, *BPI-SF* Brief Pain Inventory–Short Form, *CRP* C-reactive protein, *HbA1c* Glycated haemoglobin, *IGF-1* Insulin-like growth factor 1, *IGFBP-3* Insulin-like growth factor-binding protein 3, *IPAQ* International Physical Activity Questionnaire

For participant time lines, *see* Fig. 1. For a Standard Protocol Items: Recommendations for Interventional Trials (SPIRIT) diagram showing all data collection and the respective time points, *see* Fig. 2. All CRFs and questionnaires (in Swedish) can be obtained from the corresponding author upon request. Patients who wish to be excluded from the study will be asked if data already collected may be analysed in the study.

### Data management

All patients receive a study number, and the identification key is kept separately from the results database on a server at Sahlgrenska University Hospital. Data will be entered manually from paper CRFs into a central database on a server at Gothenburg University, where automatic back-up of server data as well as firewalls against external violation are present. A range check will be performed on applicable variables. Laboratory samples will be analysed locally. The occurrence of relevant protocol deviations will be determined and documented. Data verification and validation will be performed. When all data have been coded, validated, signed and locked, a clean file will be declared. A data manager will be responsible for the database.

### Statistical methods

The primary analyses of patient-reported physical recovery as well as sick leave, length of hospital stay and complications will be analysed on an intention-to-treat basis with generalised linear models with group as fixed effects and stratification factors as covariates. Multiplicity correction will be performed to prevent inflation of the family-wise error rate. A detailed statistical analysis plan will be finalised before any data are analysed.

### Data monitoring

Data will be continuously monitored by a research nurse to make sure that no problems have occurred with the CRFs or questionnaires.

### Harms

The intervention is already recommended to the population as a whole [51] and can be considered harmless. All complications and adverse events will be registered and analysed.

### Auditing

A research nurse will monitor the study by randomly choosing included patients to assess at site visits.

### Dissemination policy

In agreement with the internationally accepted guidelines for authorship (International Committee of Medical Journal Editors), the members of the planning group who are active in planning, running, analysing and writing will be part of the writing committee. Publication of results will be in peer-reviewed scientific journals. Results will also be communicated through patient organisations and the media as well as at professional meetings.

## Discussion

Pre-operative lifestyle interventions are receiving increased interest because of the multiple possible positive effects of physical activity [9]. In this study, we hypothesise that patients undergoing surgery for colorectal cancer will benefit from pre-operative physical activity for several reasons. Firstly, the patients are generally old, and a high proportion of the patients can be suspected to be physically active to a lesser degree than recommended [52], thereby having lower endurance capacity, strength and functional capacity than active individuals. Patients with poor functional capacity have a higher risk of a poor post-operative recovery [53]. Secondly, in parallel with their age, more of these patients can also be expected to have other diagnoses for which physical activity shows evidence of positive effects, such as hypertension, diabetes mellitus and hyperlipidaemia as well as established cardiovascular disease [9]. In addition, receiving a cancer diagnosis usually affects the mental well-being of the patient, and physical activity may possibly distract the patient’s thoughts from the disease because it has established positive effects on stress-related and mental health [9]. A further possible effect could be an increased sense of control as in ‘I do what I can to improve the outcome of the treatment’. Finally, there may be positive effects on the immune system that would be beneficial for post-operative recovery [10].

This is the first study of prehabilitation before colorectal surgery primarily aiming to include estimation of clinically relevant outcomes such as physical recovery, sick leave, hospital length of stay and adverse events. All patients in Sweden are covered by a national health insurance programme administered by the Swedish Social Insurance Agency. This, combined with accessible and reliable data on sick leave, makes length of sick leave/return to work one valuable measurement of recovery. Because almost 75% of patients with colorectal cancer have reached retirement age, another measure of physical recovery that reflects the effect of the intervention in the entire population is our primary outcome measure.

Authors of two recent systematic reviews of prehabilitation with physical activity prior to major abdominal surgery and colorectal cancer surgery have concluded that high-quality, adequately powered RCTs are needed in this field with the aim of assessing post-operative recovery, morbidity and mortality [18, 20]. They have also suggested other features, which are included in our study, namely:

- A study size of at least 400 patients [18]
- Prehabilitation combined with the ERAS protocol and use of the Clavien-Dindo classification system [20]
- QoL as an outcome measure [18]
- An intervention that is performed in a time span that corresponds to current lead times for cancer treatment (<4 weeks in the United Kingdom) [18]
- An intervention that is tolerable to patients with colorectal cancer and suitable and effective for older patients (>75 years old) [18]

Currently, there are at least six other ongoing trials of prehabilitation including physical activity before surgery for colorectal cancer registered with ClinicalTrials.gov. One is a pilot study in Canada assessing the effect of physiatry with several clinically relevant outcome measures as secondary endpoints, aiming to enrol 70 patients (ClinicalTrials.gov identifier: NCT02531620). Physiatrists are physicians who are medical experts in maximising a patient’s overall ability to function well and live independently. Another Canadian RCT compares supervised physical activity combined with nutrition counselling and relaxation strategies before and after surgery with rehabilitation, with the 6-minute walk test as a primary outcome measure (ClinicalTrials.gov identifier: NCT02586701). The same researchers also have a small active trial with protein synthesis as the primary outcome measure (ClinicalTrials.gov identifier: NCT01919541) as well as an RCT in which they plan to enrol 120 patients with the aim of determining if a programme of physical activity, nutritional supplementation and relaxation techniques is effective at decreasing post-operative complications in patients with colorectal cancer, with complications assessed within 30 days as the primary outcome measure (ClinicalTrials.gov identifier: NCT02502760). In a trial in Switzerland with the aim of determining whether a combined cardiorespiratory and strengthening training prior to colorectal surgery decreases the rate of surgery-related complications, comprehensive complication index within 30 days is the primary outcome measure, and the researchers plan to enrol 112 patients (ClinicalTrials.gov identifier: NCT02746731). The aim of a Norwegian study is to investigate whether pre-operative exercise training including pelvic floor muscle training during pre-operative radiotherapy can reduce symptoms of bowel, urinary and sexual dysfunction after low anterior resection for rectal cancer (ClinicalTrials.gov identifier: NCT02538913).

The nature of the intervention in our trial is simple and would be easy to implement in clinical practice if shown to be effective. This is important because the compliance rate of any given intervention is crucial for its efficacy. The reason for failure of more intensive physical activity interventions [14] may be lack of compliance. Thus, we chose a physical activity intervention level with the aim of achieving a high compliance rate.

The post-operative intervention in this study is similar to the recommendations on physical activity given to the general public [54]. This is practical for several reasons. On one hand, recommendations to the general public convey few side effects for most individuals, which makes the intervention safe also for patients with colorectal cancer. On the other hand, the intervention is known to have several benefits other than the possible benefits analysed in this study [54]. One expectation is that a portion of the participants in the study who change their lifestyle in connection with a life-changing event such as cancer treatment will continue with their new lifestyle after the study period and thus have several other health benefits.

## Trial status

Inclusion of patients started in January 2015 and is ongoing.

## Additional file

Additional file 1: SPIRIT checklist listing where important information on the study can be found in this manuscript. (PDF 377 kb)

## Abbreviations

ASA: American Society of Anesthesiologists physical status
AUDIT-C: Alcohol Use Disorders Identification Test alcohol consumption questions
BPI-SF: Brief Pain Inventory–Short Form
CRF: Case report form
CRP: C-reactive protein
ERAS: Enhanced Recovery After Surgery
Hb: Haemoglobin
HbA1c: Glycated haemoglobin
IGF-1: Insulin-like growth factor 1
IGFBP-3: Insulin-like growth factor-binding protein 3
IMT: Inspiratory muscle training
IPAQ: International Physical Activity Questionnaire
MIP: Maximal inspiratory pressure
QALY: Quality-adjusted life-years
QoL: Quality of life
RCT: Randomised controlled trial
SPIRIT: Standard Protocol Items: Recommendations for Interventional Trials
WBC: White blood cells
