# Correction to: The effect of pre- and post-operative physical activity on recovery after colorectal cancer surgery (PHYSSURG-C): study protocol for a randomised controlled trial

**DOI:** 10.1186/s13063-020-04979-8

**Published:** 2020-12-23

**Authors:** Aron Onerup, Eva Angenete, David Bock, Mats Börjesson, Monika Fagevik Olsén, Elin Grybäck Gillheimer, Stefan Skullman, Sven-Egron Thörn, Eva Haglind, Hanna Nilsson

**Affiliations:** 1 Scandinavian Surgical Outcomes Research Group (SSORG), Department of Surgery, Institute of Clinical Sciences, Sahlgrenska Academy, University of Gothenburg, Gothenburg, Sweden; 2 Department of Neuroscience and Physiology, Sahlgrenska Academy, University of Gothenburg, Gothenburg, Sweden; 3 Department of Food and Nutrition and Sport Science, University ofGothenburg, Gothenburg, Sweden; 4 Sahlgrenska University Hospital/Östra, Gothenburg, Sweden; 5 Department of Gastrosurgical Research and Education, Sahlgrenska Academy at Gothenburg University, Gothenburg, Sweden; 6 Department of Physical Therapy and Surgery, Sahlgrenska University Hospital, Gothenburg, Sweden; 7 Department of Surgery, Skövde Hospital/KSS, Skövde, Sweden; 8 Department of Anesthesiology, Institute of Clinical Sciences, Sahlgrenska Academy, University of Gothenburg, Gothenburg, Sweden

**Correction to: Trials (2017) 18:212**

**https://doi.org/10.1186/s13063-017-1949-9**

Following publication of the original article [1], we were notified of two errors in the Methods section.

- In the ”Allocation” section, the sentence:

*”To ensure good balance of participant characteristics in each group, randomisation will be stratified with regard to surgical method (laparoscopic* versus *open), tumour site and pre-operative treatment (colon, rectum with/without radiotherapy), and study centre.”* However, study centre was not included in the electronic randomisation system as stratification.

Should instead read:

*”To ensure good balance of participant characteristics in each group, randomisation will be stratified with regard to surgical method (laparoscopic* versus *open) and tumour site and pre-operative treatment (colon, rectum with/without radiotherapy).”*

- In the ”Sample size” section, there was a mix of two approaches considered, which is reflected in the fact that we wrote both that we needed 640 evaluable patients and that this would require 640 randomised patients to account for losses to follow-up. We planned to include 538 evaluable patients, which would result in a need to randomize 640 patients to account for patients being lost to evaluation, as found in the first 100 patients and the interim analysis. Initially we included a Bonferroni correction due to second look into the calculation but discarded this since no statistical testing was performed during the interim analysis. We therefore planned to recruit 538 evaluable participants.

Therefore the sentence:

*”For true rates of these magnitudes, a difference will be detected with 80% power using a total of 640 patients and a two-sided test with a 2.5% significance level (Bonferroni adjustment for interim look).”*

Should instead read:

*”For true rates of these magnitudes, a difference will be detected with 80% power using a total of 538 patients and a two-sided test with a 5% significance level.”*
